# Simultaneous analysis of tumor-infiltrating immune cells density, tumor budding status, and presence of lymphoid follicles in CRC tissue

**DOI:** 10.1038/s41598-022-26225-8

**Published:** 2022-12-16

**Authors:** Adam R. Markowski, Anna J. Markowska, Wiktoria Ustymowicz, Anna Pryczynicz, Katarzyna Guzińska-Ustymowicz

**Affiliations:** 1 Department of Internal Medicine and Gastroenterology, Polish Red Cross Memorial Municipal Hospital, 79 Henryka Sienkiewicza Street, 15-003 Bialystok, Poland; 2 Medical University of Bialystok, 2C Adama Mickiewicza Street, 15-222 Bialystok, Poland; 3 Medical University of Warsaw, 61 Żwirki i Wigury Street, 02-091 Warsaw, Poland; 4 Department of General Pathomorphology, Medical University of Bialystok, 13 Jerzego Waszyngtona Street, 15-269 Bialystok, Poland

**Keywords:** Gastrointestinal diseases, Biomarkers, Pathogenesis

## Abstract

Colorectal cancer (CRC) affects more than 1,000,000 people worldwide each year. Recently, the number of young patients with early-onset colorectal cancer has increased, and right-sided colorectal cancer is still often diagnosed only in advanced stages. The TNM classification is not perfect for CRC staging. This study aimed to perform, for the first time, simultaneous analysis of tumor-infiltrating immune cell density, presence of lymphoid follicles, and budding status in CRC tissue. Intraoperative samples of neoplastic tissue were collected from 195 consecutive patients who were admitted to the surgical ward for elective colorectal surgery. Histological parameters were assessed in the tissue samples: tumor budding foci, poorly differentiated clusters and areas of poorly differentiated components. Tumor-infiltrating immune cells (tumor-associated neutrophils and tumor-infiltrating lymphocytes) were detected in five randomly chosen, areas at the tumor center and at the invasive front. Additionally, the presence of lymphoid follicles in CRC tissue was assessed. Tumor budding parameters were positively correlated with colorectal cancer advancement or histologic (mucinous) type of CRC. The number of poorly differentiated clusters was higher in younger patients. Lower densities of CD3 and CD4 lymphocytes were seen in CRC with a greater depth of tumor invasion. Lower densities of CD3 and CD8 lymphocytes were found in CRC with metastases to the surrounding lymph nodes. The lower density of CD8 lymphocytes was observed in CRC with distant metastases. Lower densities of tumor-associated neutrophils and tumor-infiltrating lymphocytes (CD3 and CD8) were revealed in CRC without lymphoid follicles. The number of lymphoid follicles was higher in patients with less advanced CRCs. Three histopathology markers, such as high tumor budding, scanty lymphocyte infiltration, and the poverty of lymphoid follicles, complement each other, appear to be reliable indicators of colorectal cancer progression, and could be useful in everyday medical practice, but their widespread use requires further research. We propose to take into account these markers, in the assessment of colorectal cancer advancement, in addition to the TNM classification.

## Introduction

Colorectal cancer (CRC) is the third most commonly diagnosed malignancy and the fourth leading cause of cancer-related death in the world^1^. For this reason, CRC is a consistent topic of interest to scientists as they try to determine more about its development^2^.

CRC represents a diversified group of tumors that display miscellaneous clinical and pathological features. The prognosis of CRC patients usually depends on the stage when the cancer is detected and treatment is started. The TNM classification provides strong prognostication for patients with early and late disease. However, there are differences in the course of the intermediate stages of CRC^3^, suggesting a complex and not fully recognized interaction of ultimately unidentified factors. Knowing them can help identify patients at high risk of aggressive progression, formation of metastases, and recurrence.

Recently, unfavorable trends have been observed in the global incidence of CRC. Marked increases in both CRC incidence and mortality are observed in many low-income and middle-income countries; stabilizing or decreasing trends tend to be seen only in countries with the highest Human Development Index^1^. CRC, especially right-sided, is still often diagnosed in advanced stages; screening programs can change this relationship^4^. Particularly worrisome changes are observed in the younger age group, as the recent annual increase in the incidence of CRC in people under the age of 65 has slightly increased by 1–2% annually^5^. Understanding all aspects of CRC progression will help to develop a new approach to early diagnosis and cancer therapy. Some histological features may be important in the treatment decision of CRC patients.

Most recently, in early CRC, according to current endoscopic guidelines, intermediate or high-grade tumor budding at the site of deepest invasion is one of several risk factors for lymph node metastasis^6^. Tumor-infiltrating immune cells are often interpreted as the host protecting against tumor development and play a key role in inhibiting tumor invasion and metastasis^7^. Assessing some tumor features can help identify patients with low- and intermediate-stage CRC at risk of a poorer prognosis.

The objective of this study was to perform, for the first time, simultaneous analysis of tumor-infiltrating immune cell density, the presence of lymphoid follicles, and budding status in CRC tissue.

## Materials and methods

### Patients

This study enrolled 195 consecutive patients who were admitted to the surgical ward for elective colorectal surgery. This was a retrospective analysis of prospectively collected data, conducted at a teaching hospital. Pre-treatment diagnosis of CRC was evaluated based on colonoscopy with biopsy and CT scan. All patients gave their written informed consent for participation in the study.

The study was in line with the principles outlined in the Declaration of Helsinki and approved by the Ethical Committee for Human Studies of the Medical University of Bialystok, Poland, ethics committee approval no R-I-002/228/2018. Informed consent was obtained from all subjects involved in the study.

### Histological analysis

Tumor tissue samples were obtained intraoperatively. The dissected tissues were immediately placed into liquid nitrogen and transferred for histological evaluation. Pathologic analysis using a standardized reporting template was performed on the resected specimens and staged according to the American Joint Committee on Cancer criteria, version 8 guidelines. The patients were classified according to the TNM staging (tumor, nodes, metastasis). Microscopic examination of the postoperative material was performed on formalin-fixed and paraffin-embedded sections. Hematoxylin and eosin-stained serial sections of the primary tumor were chosen for typical histological analysis and for immunohistochemical studies after selection of representative areas of the tumor.

### Tumor budding parameters analysis

Tumor budding is a histological feature diagnosed at high magnification and defined as single cells or clusters of neoplastic cells at the invasive tumor front^8^.

**Figure 1:**
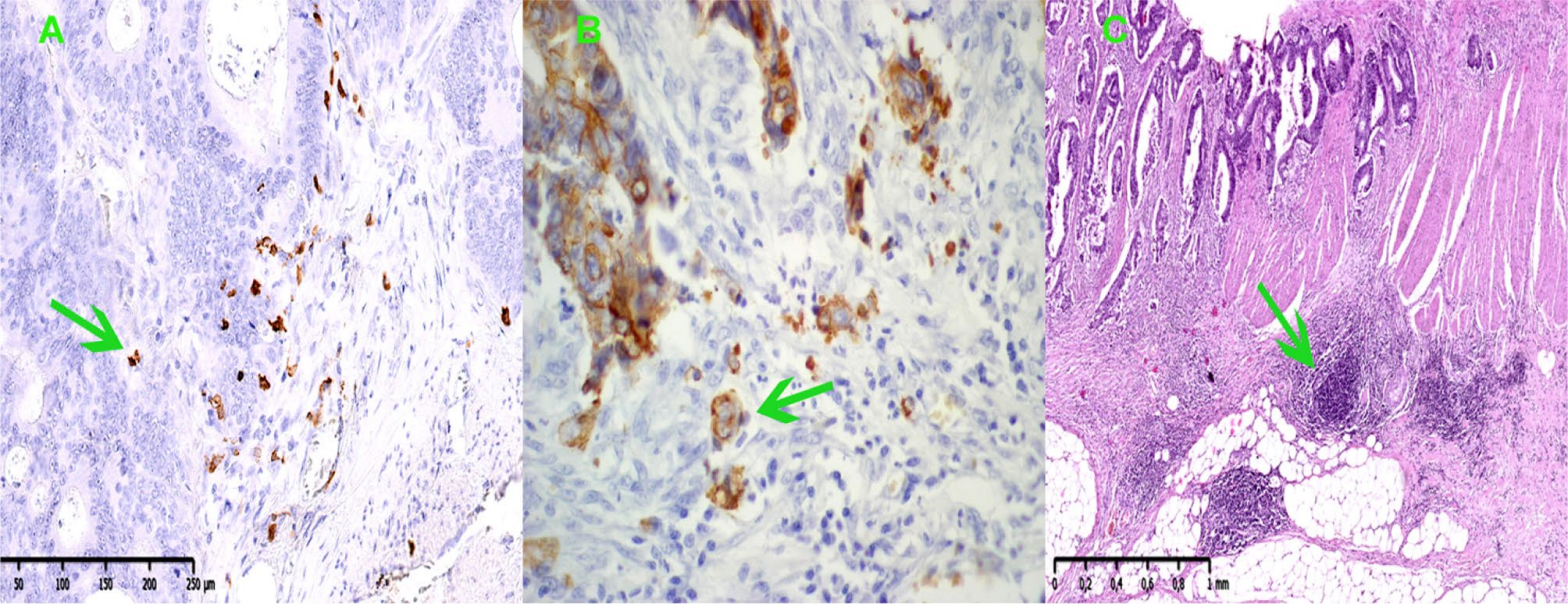
(**A**) Representative examples of CD8 cells expression in colorectal cancer tissue, × 100 magnification. (**B**) Representative examples of tumor budding at the front of invasion in colorectal cancer tissue, × 40 magnification. (**C**) Representative examples of lymphoid follicles in the front of tumor invasion in colorectal cancer tissue, × 40 magnification.

Tumor budding foci (TBFs) were defined as isolated cancer cells or clusters of ≤ 4 cells in the stroma at the invasive margin using H&E staining and cytokeratin immunohistochemical staining, Fig. 1B. The number of TBFs in the invasive frontal region was counted within the field of densest budding of 0.785 mm2 at 20 × magnification and graded according to its number as low (TBF-1): 0–4 buds, intermediate (TBF-2): 5–9 buds, and high-grade (TBF-3): ≥ 10 buds^9^. The invasive front is a 1-mm region centered on the border separating the malignant cell nests from the host tissue^10^. The tumor center represents the remaining tumor area inside the invasive front and the peritumor area corresponds to tissue outside of the invasive front. The invasive front of colorectal cancer represents a dynamic interface between pro- and antitumor factors^11^. The infiltrating tumor border configuration and tumor budding promote the progression and dissemination of tumor cells by penetrating the vascular and lymphatic vessels, and the host attempts to fend off this attack by mounting an immune response using cytotoxic T lymphocytes.

Poorly differentiated clusters (PDCs) were defined as cancer cell clusters comprising ≥ 5 cancer cells infiltrating the stroma and lacking gland formation^12^. The number of poorly differentiated clusters (PDCs) in the invasive frontal region was graded as low PDC-1 (0–4 PDC), moderate PDC-2 (5–9 PDC) and severe PDC-3 (≥ 10 PDC).

Areas of poorly differentiated components (PORs) were defined as regions where the cancer had no glandular formation^13^. The lowest magnification of the objective lens for which the poorly differentiated component filled the field of vision was regarded as the extent of the poorly differentiated component of CRC. POR was graded as low POR-1 (when POR did not occupy the field with a × 40 objective lens), moderate POR-2 (when POR did not occupy the greater part of the tumor) and severe POR-3 (when POR occupied the greater part of the tumor).

### Immunohistochemical analysis of tumor-infiltrating immune cells

Tissue sections were stained by immunohistochemistry according to the XT-ultraView-DABv3 procedure. Tumor-infiltrating lymphocytes (TILs) and tumor-associated neutrophils (TANs) were assessed in tissue material stained with hematoxylin and eosin, and evaluated by a pathologist who was blinded to any patient clinical data, Fig. 1A. At the same time, the tissues were assessed for the presence of lymphoid follicles, Fig. 1C. To identify the types of lymphocytes, the following three antibodies were used: anti-CD3 (2GV6) rabbit monoclonal primary antibody (Ventana), anti-CD4 (SP35) rabbit monoclonal primary antibody (Ventana), anti-CD8 (SP57) rabbit monoclonal antibody (Ventana). Staining was performed automatically on the Benchmark XT using Ventana’s proprietary protocols. The histopathological assessment was performed using an Olympus CX21 light microscope at 400 × magnification. Tumor-infiltrating immune cells (TIICs) were detected in five randomly chosen, nonadjacent, nonoverlapping areas separately at the tumor center (TC) and at the invasive front (IF) to ensure uniformity and representativeness of the obtained results. Tumor-infiltrating immune cells were counted in 5 fields of view [FOV], the number of cells was calculated on 0.785 mm^2^ of the tissue surface, and the result was the mean value [cells/FOV].

### Statistical analysis

Statistical analysis was performed with Statistica 13.3 (TIBCO Software Inc., Palo Alto, California, USA). The normality of the data was verified with Shapiro–Wilk Test. Levene’s test was used to check for homogeneity of variances. Spearman's rank-order correlation coefficient was used to measure the strength of a relationship between variables. Statistical differences between two groups were determined using the Mann–Whitney *U* test. Statistical differences between multiple groups were determined using the Kruskal–Wallis test followed by Dunn’s post hoc test for multiple comparisons. Statistical significance for all test was assumed if a p‐value was less than 0.05.

## Results

### Characteristics of the population

Of the 195 patients, 40.51% (n = 79) were female, Table 1. All patients were white. The mean age of CRC patients at diagnosis was 68 years (range 27–89). The largest group consisted of patients aged 70–79 years (42.57%), and the smallest group had those aged 80 years and above (9.23%). The rectum was the part of the large intestine where cancer was most common. Mucinous adenocarcinoma occurred in 36.93% of cases. There was no undifferentiated cancer (G4), the least frequent was well-differentiated cancer (low grade, G1) (n = 1, 0.51%), and the most common was moderately differentiated cancer (medium grade, G2) (n = 172, 88.21%). The group with poorly differentiated cancer (high grade, G3) included 22 patients. The depth of tumor invasion varied; the least frequent were grade T1 (< 3%) and T4 (< 6%), and the most common grade was T3 (78%). Most patients had no evidence of lymph node involvement (57.95%) or distant metastases (73.85%). The number of patients in each group according to TNM classification was as follows: n = 26 (13.33%, TNM-I), n = 64 (32.83%, TNM-II), n = 54 (27.69%, TNM-III), n = 51 (26.15%, TNM-IV). Neural invasion was found in almost a quarter of cases (23.59%), and vascular invasion was found in almost half of cases (55.9%).

**Table 1:** Clinical characteristics of the study group (n = 195).

Gender	Male, n = 116 (59.49%)
	Female, n = 79 (40.51%)
Age	< 60, n = 43 (22.05%)
	60–69, n = 51 (26.15%)
	70–79, n = 83 (42.57%)
	≥ 80, n = 18 (9.23%)
Primary tumor location	Rectum, n = 83 (42.57%)
	Sigmoid colon, n = 51 (26.15%)
	Other parts of colon, n = 46 (23.59%)
	Cecum, n = 15 (7.69%)
Histologic type	Adenocarcinoma n = 123 (63.07%)
	Mucinous adenocarcinoma n = 72 (36.93%)
Histologic grade	G1, well‐differentiated, low grade, n = 1 (0.51%)
	G2, moderately differentiated, n = 172 (88.21%)
	G3, poorly differentiated, high grade, n = 22 (11.28%)
	G4, undifferentiated, high grade, n = 0
T stage	T1, submucosa, n = 5 (2.56%)
	T2, muscularis propria, n = 27 (13.85%)
	T3, subserosa, n = 152 (77.95%)
	T4, serosa or other organs, n = 11 (5.64%)
N stage	N0, absent, n = 113 (57.95%)
	N1, present, n = 82 (42.05%)
M stage	M0 absent, n = 144 (73.85%)
	M1 present, n = 51 (26.15%)
TNM stage	TNM-I, n = 26 (13.33%)
	TNM-II, n = 64 (32.83%)
	TNM-III, n = 54 (27.69%)
	TNM-IV, n = 51 (26.15%)
Lymphovascular invasion	LVI-0, absent, n = 86 (44.1%)
	LVI-1, present, n = 109 (55.9%)
Perineural invasion	PNI-0, absent, n = 149 (76.41%)
	PNI-1, present, n = 46 (23.59%)

### Tumor budding parameters

In the studied population of colorectal cancer patients, tumor budding parameters (TBPs) were evaluated, Table 2. The mean number of buds was 3.47 ± 0.23, but low-grade TBPs represented a majority. The severity of CRC malignancy based on the number of tumor budding foci was high (TBF-3) in 5.64%, medium (TBF-2) in 18.97%, and low (TBF-1) in 75.39% of patients. The count of poorly differentiated cancer cell clusters was high (PDC-3) in 18.04%, medium (PDC-2) in 35.57%, and low (PDC-1) in 46.39% of cases. A high grade of the poorly differentiated component (POR-3) was shown in 17.53%, medium-grade (POR-2) in 34.02%, and low-grade (POR-1) in 48.45% of patients. We compared these parameters with each other and with the severity of CRC malignancy according to the TNM classification.

**Table 2:** Tumor budding parameters in colorectal cancer tissue.

Tumor budding foci	TBF-1 (75.39%)
	TBF-2 (18.97%)
	TBF-2 (18.97%)
Poorly differentiated clusters	PDC-1 (46.39%)
	PDC-2 (35.57%)
	PDC-3 (18.04%)
Areas of poorly differentiated components	POR-1 (48.45%)
	POR-2 (34.02%)
	POR-3 (17.53%)

PDC was positively correlated with TBF (r = 0.23), and especially with POR (r = 0.87), Table 3. An analysis also showed an evident change in tumor budding status with the progression of the disease. Budding parameters were positively correlated with colorectal cancer advancement. TBPs were positively correlated with CRC grading (G): TBF (r = 0.35), PDC (r = 0.24) and POR (r = 0.25), Table 4.

**Table 3:** Spearman's rank correlation coefficients between selected tumor budding parameters.

	TBF	PDC	POR
TBF	1.000	0.230*	0.227*
PDC	0.230*	1.000	0.866*
POR	0.227*	0.866*	1.000

Marked correlation coefficients are significant with p < 0.05.

**Table 4:** Spearman's rank correlation coefficients between selected tumor budding parameters and clinicopathological parameters.

	Age	Sex	Localis	G	T	N	M	TNM
TBF	− 0.002	0.010	0.100	0.354*	0.115	0.127	0.813*	0.678*
PDC	− 0.063	− 0.074	0.024	0.236*	0.093	0.122	0.220*	0.198*
POR	− 0.030	− 0.092	0.064	0.250*	0.059	0.108	0.202*	0.189*

Marked correlation coefficients are significant with p < 0.05.

Moderately differentiated CRCs (G2) had lower TBPs than poorly differentiated (G3) cancers, TBF (p < 0.0001), PDC (p = 0.005) and POR (p = 0.003). There was no direct correlation between TBPs and T stage (T1, T2, T3, T4) or N stage (N0, N1, N2), but some relationships were also statistically significant in these cases. Node-negative patients (N0) had a lower PDC and POR than patients with the highest stage (N2) of lymph node involvement (p = 0.02 and p = 0.03, respectively). Some correlations were also found between TBPs and the presence of distant metastases (M-stage): PDC (r = 0.22) and POR (r = 0.20), but it was especially strong for TBF (r = 0.81), Table 4. CRC patients with distant metastases (M1) had higher TBPs than CRC patients without metastases (M0): TBF (p < 0.0001), PDC (p = 0.004) and POR (p = 0.012). The above relationships translated into correlations between the severity of TBPs and CRC advancement according to the TNM classification: TBF (r = 0.68), PDC (r = 0.19) and POR (r = 0.17). Low-stage CRCs (TNM-I and TNM-II) had lower TBPs than the high-stage (TNM III-IV) CRC: TBF (p < 0.0001, p < 0.0001), PDC (p = 0.001, p = 0.005), POR (p = 0.008, p = 0.025), respectively. Within high-stage cancers (TNM-III and TNM-IV), only one TBP was correlated, so TBF was lower in TNM-III CRC than in TNM-IV CRC (p < 0.0001). Some relationships were also statistically significant for lymphovascular invasion (LVI), and LVI-negative CRC patients had a lower PDC and POR than LVI-positive CRC patients (p = 0.048 and p = 0.008, respectively). Additionally, histologic type was positively correlated with TBF (r = 0.27), and TBF was higher in mucinous adenocarcinomas than in nonmucinous adenocarcinomas (p = 0.027). There were no statistically significant correlations between TBPs and sex in CRC patients, but PDC was higher in younger patients (p = 0.038).

Combining the above information, it can be concluded that low TBPs were observed in cases of low-advanced cancer assessed by several different criteria: histologic type, histologic grade, lymphovascular invasion, regional lymph node metastases, distant metastases, and TNM stage.

### Tumor-infiltrating immune cells and lymphoid follicles

The number of tumor-infiltrating immune cells was presented as the mean number ± standard error of the mean (M ± SE) in one field of view (FOV): CD8-TC = 59.61 ± 6.28, CD8-IF = 185.73 ± 11.13, CD4-TC = 28.60 ± 2.18, CD4-IF = 122.81 ± 7.64, CD3-TC = 33.73 ± 3.11, CD3-IF = 131.37 ± 9.56, TAN-TC = 52.27 ± 4.53, TAN-IF = 82.43 ± 8.29. Lymphoid follicles (LFs) were found in 47.7% patients. When the numbers of all evaluated lymphocytes were compared at two different locations within one tumor, i.e., in the tumor center and in the invasive front (TC vs IF), other relationships were found. A decidedly higher combined number of all evaluated lymphocytes was found in the invasive front than in the tumor center (p < 0.0000), and the number of tumor-associated neutrophils in the invasive front (TAN-IF) was also higher than that in the tumor center (TAN-TC) (p = 0.0015). Of the 4 types of tumor-infiltrating immune cells (TIICs) evaluated, the highest density was found for CD8 lymphocytes in the invasive front (CD8-IF), while the lowest density was found for CD4 lymphocytes in the tumor center (CD4-TC).

**Figure 2:**
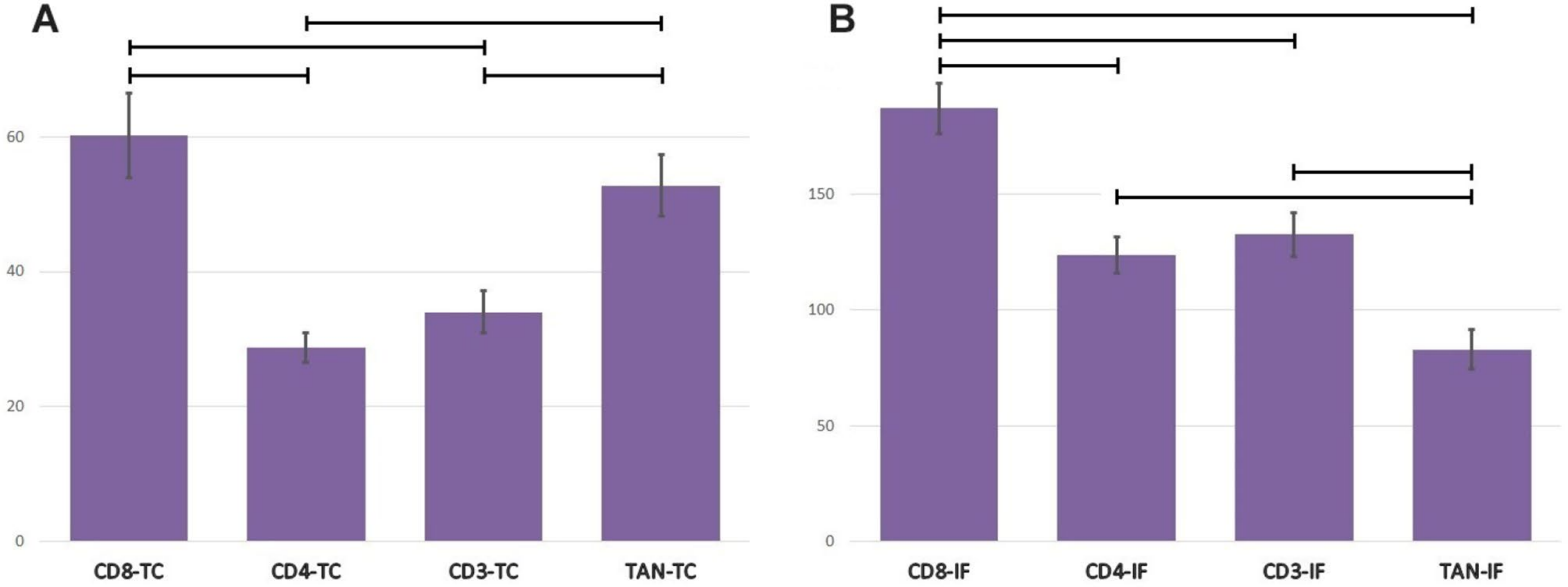
(**A**) Tumor-infiltrating immune cells density in tumor center. Data are presented as means and standard errors. Horizontal lines indicate significant differences at p < 0.05. (**B**) Tumor-infiltrating immune cells density in invasive front. Data are presented as means and standard errors. Horizontal lines indicate significant differences at p < 0.05.

When analyzing the distribution of individual immune cells within one tumor, we noticed that the number of tumor-infiltrating immune cells in the tumor center (TIIC-TC) was not the same for all cell types. In the tumor center, the mean numbers of the two cell types (CD8-TC and TAN-TC) were highest and did not differ statistically from each other (CD8-TC vs TAN-TC; p = 0.69), Fig. 2A. Similarly, the mean numbers of the other two cell types (CD4-TC and CD3-TC) were the lowest, did not differ statistically from one another (CD4-TC vs CD3-TC; p = 0.87), and simultaneously were lower than the first two (CD4-TC vs CD8-TC; p < 0.0001. CD4-TC vs TAN-TC; p = 0.0018. CD3-TC vs CD8-TC; p = 0.0005. CD3-TC vs TAN-TC; p < 0.026).

The number of tumor-infiltrating immune cells in the invasive front was also not the same for all cell types. In the invasive front, the mean number of one cell type (CD8-IF) was higher than the others (p < 0.0001), and the numbers of one cell type (TAN-IF) was lower than all others: TAN-IF vs CD8-IF (p < 0.0001), TAN-IF vs CD4-IF (p < 0.024), TAN-IF vs CD3-IF (p < 0.003). The numbers of the other two cells (CD4-IF and CD3-IF) had intermediate values and did not differ statistically from each other (p = 0.93), Fig. 2B.

During the analysis of individual TNM components, further changes were found. A negative correlation was found between the presence of metastases and the density of CD8 lymphocytes in the invasive front, Table 5.

**Table 5:** Spearman's rank correlation coefficients between selected tumor-infiltrating immune cells and clinicopathological parameters.

	CD8 TC	CD8 IF	CD4 TC	CD4 IF	CD3 TC	CD3 IF	TAN TC	TAN IF
Age	0.009	− 0.039	0.035	− 0.051	0.098	0.057	− 0.046	0.016
Sex	− 0.005	− 0.040	− 0.061	− 0.018	− 0.099	− 0.164*	− 0.023	0.032
Location	0.187*	0.136	− 0.073	− 0.097	0.089	0.095	0.147*	0.027
G	0.022	0.071	0.108	0.028	0.015	0.026	0.022	0.134
T	− 0.098	− 0.037	− 0.172*	− 0.090	− 0.172*	− 0.219*	− 0.120	− 0.095
N	− 0.184*	− 0.123	− 0.096	− 0.045	− 0.020	− 0.154*	− 0.093	− 0.045
M	− 0.053	− 0.142*	0.069	− 0.026	0.018	0.025	− 0.084	0.009
TNM	− 0.040	− 0.064	− 0.003	− 0.060	− 0.071	− 0.045	− 0.101	− 0.052

Marked correlation coefficients are significant with p < 0.05.

**Figure 3:**
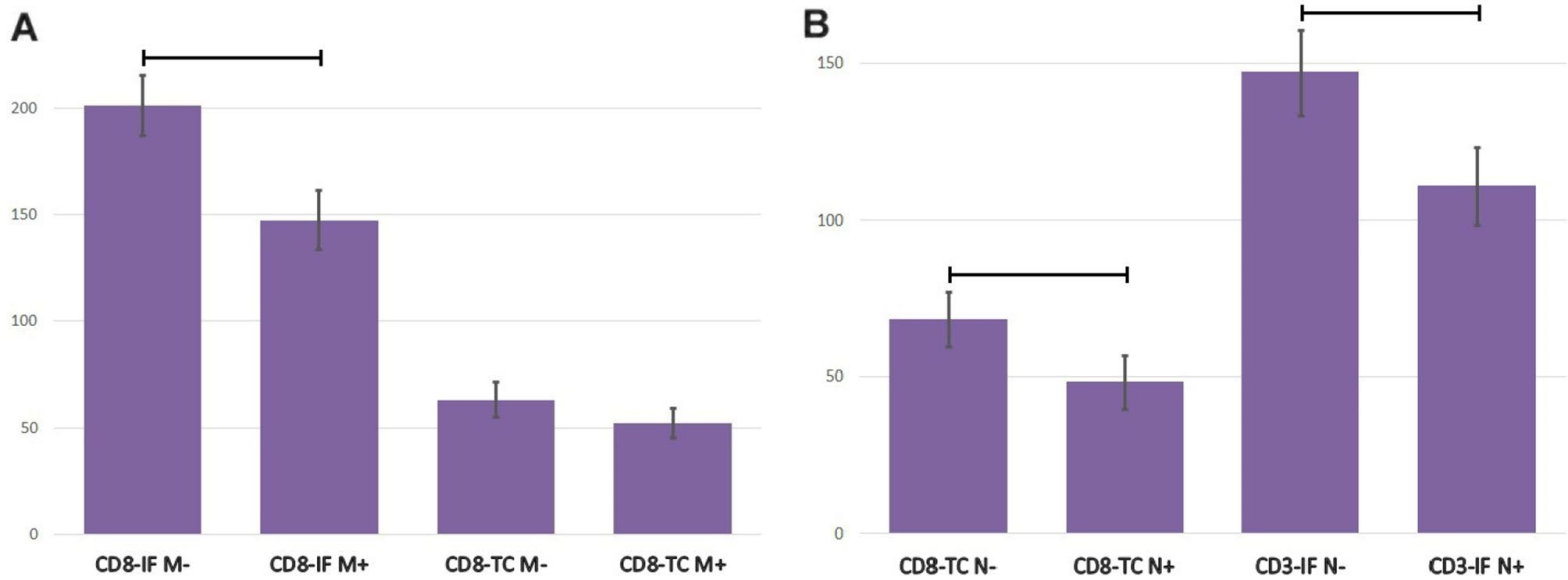
(**A**) The densities of tumor-infiltrating lymphocytes CD8 in invasive front and tumor-associated neutrophils in tumor center in colorectal cancer tissue of patients with distant (M+) and no metastases (M−). Data are presented as means and standard errors. Horizontal lines indicate significant differences at p < 0.05. (**B**) The densities of tumor-infiltrating lymphocytes CD8 in tumor center and CD3 in invasive front in colorectal cancer tissue with (N+) and without (N−) metastases to the surrounding lymph nodes. Data are presented as means and standard errors. Horizontal lines indicate significant differences at p < 0.05.

A lower number of CD8 lymphocytes was found in the invasive front of CRC with confirmed distant metastases than in nonmetastatic tumors (147.39 ± 13.76 vs. 201.26 ± 14.20; p = 0.025), Fig. 3A. A similar trend was observed in the tumor center, as the number of CD8 cells was lower in tumors with confirmed nodal metastases (52.27 ± 6.85 vs. 63.10 ± 8.19); unfortunately, this difference did not reach statistical significance (p = 0.067), Fig. 3A.

A lower number of CD8 lymphocytes was found in the tumor center of CRC with metastases to the surrounding lymph nodes than in nonmetastatic tumors (48.28 ± 8.57 vs. 68.44 ± 8.80; p = 0.005). The same relationship was observed for CD3 lymphocytes in the invasive front (110.92 ± 12.46 vs. 147.11 ± 13.65; p = 0.037), Fig. 3B.

**Figure 4:**
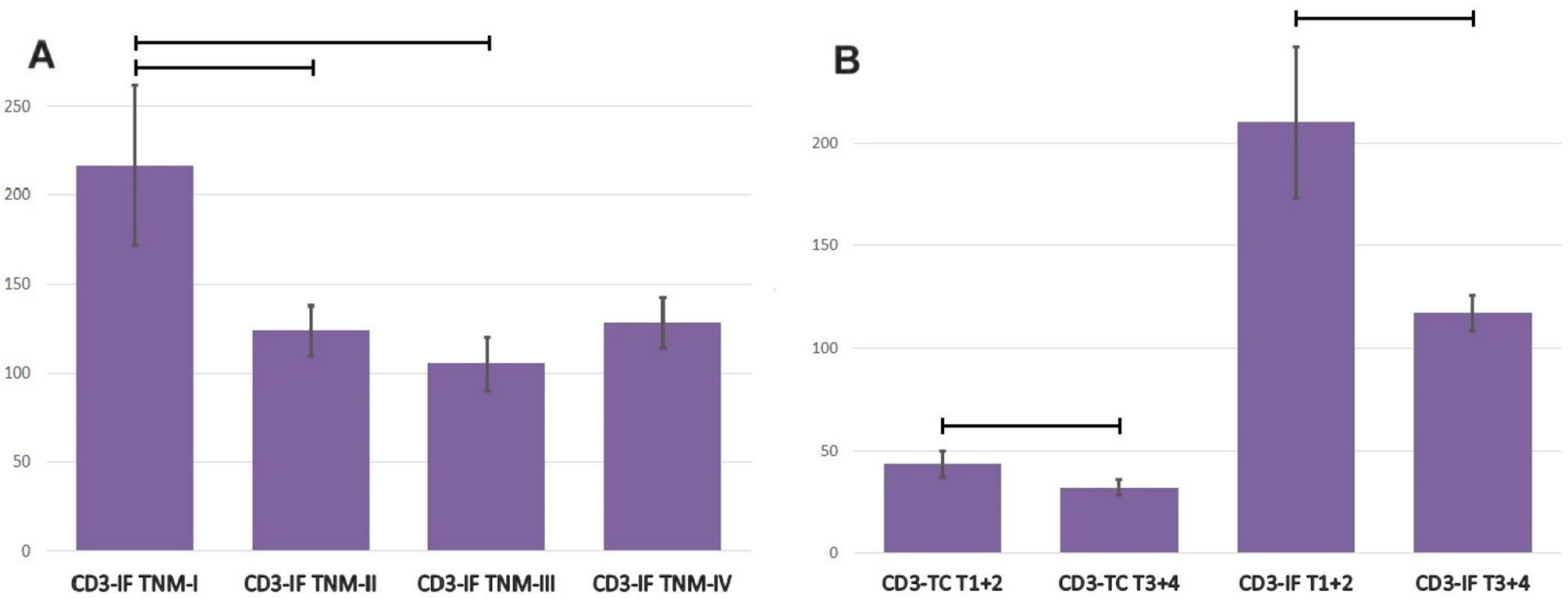
(**A**) The densities of tumor-infiltrating lymphocytes CD3 in invasive front in colorectal cancer tissue of patients with different stages of cancer according to TNM classification. Data are presented as means and standard errors. Horizontal lines indicate significant differences at p < 0.05. (**B**) The densities of tumor-infiltrating lymphocytes CD3 in invasive front and tumor center in colorectal cancer tissue of patients with different depth of cancer invasion, divided into two groups (T1 + T2 vs T3 + T4). Data are presented as means and standard errors. Horizontal lines indicate significant differences at p < 0.05.

Statistical analysis also revealed changes in TIICs density depending on the stage of colorectal cancer according to TNM-8, although statistically significant changes were found only for CD3-IF (TNM-I vs TNM-II; 217.00 ± 44.68 vs. 123.84 ± 14.22; p = 0.019. TNM-I vs TNM-III; 217.00 ± 44.68 vs. 105.54 ± 14.99; p = 0.017) and CD3-TC (TNM-I vs TNM-II; 42.12 ± 7.71 vs. 30.97 ± 5.99 14.22; p = 0.019. TNM-I vs TNM-III; 42.12 ± 7.71 vs. 32.00 ± 5.43; p = 0.037), Fig. 4A. When patients were divided into two groups depending on the T-grade (T1 + 2 vs. T3 + 4), further statistically significant differences were found. In the case of a smaller extent of the primary tumor invasion (T1 + 2), a higher number of TIICs were found, for CD4-TC (35.97 ± 4.98 vs. 27.30 ± 2.42; p = 0.010), for CD3-TC (43.56 ± 6.50 vs. 32.17 ± 3.51; p = 0.002), and for CD3-IF (210.25 ± 36.93 vs. 117.18 ± 8.53; p = 0.001), Fig. 4B.

When patients were divided into two groups depending on the severity of CRC according to the TNM classification (TNM-I + II vs. TNM-III + IV), a distinctly (and statistically significant) difference was visible only for CD8-IF, and a higher number of CD8 cells was found in less advanced tumors (209.18 ± 18.24 vs. 168.31 ± 13.53; p = 0.043).

**Figure 5:**
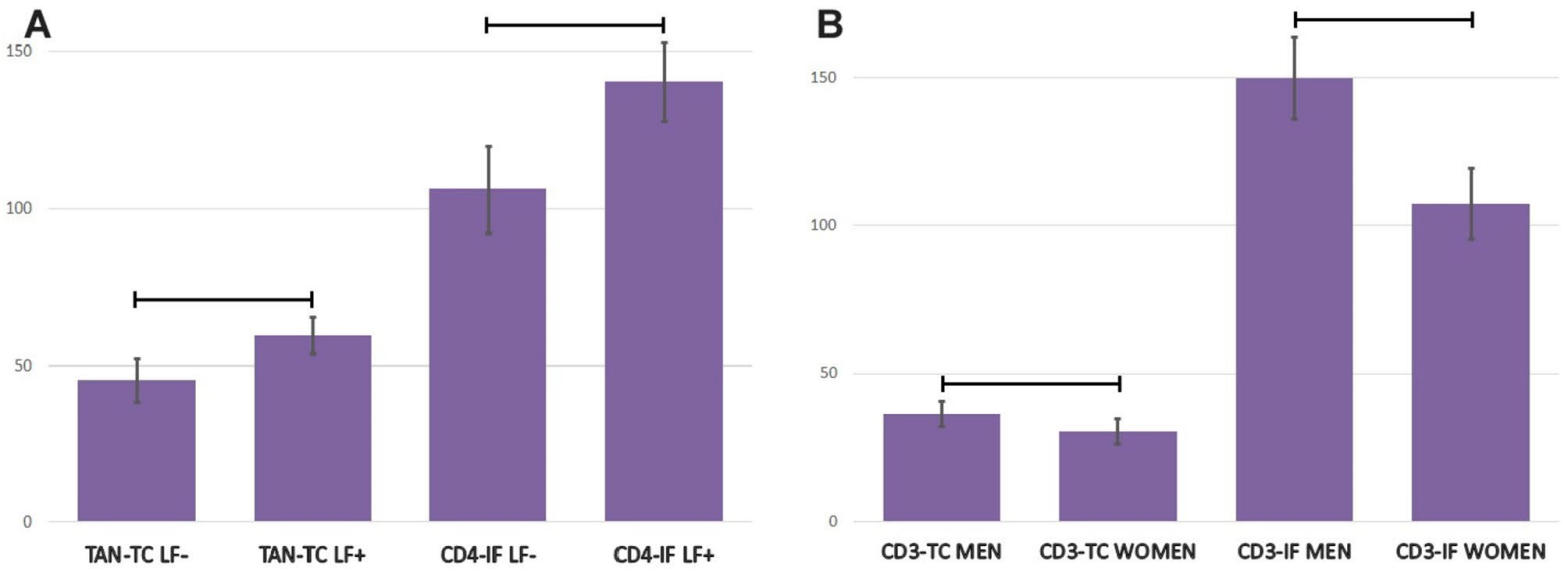
(**A**) The densities of tumor-infiltrating lymphocytes CD4 in invasive front and tumor-associated neutrophils (TAN) in tumor center in colorectal cancer tissue of patients with lymphoid follicles (LF+) and without lymphoid follicles (LF−). Data are presented as means and standard errors. Horizontal lines indicate significant differences at p < 0.05. (**B**) The densities of tumor-infiltrating lymphocytes CD3 in tumor center and invasive front in colorectal cancer tissue of male and female patients. Data are presented as means and standard errors. Horizontal lines indicate significant differences at p < 0.05.

Additionally, the presence of LFs was higher in patients with less advanced tumors (TNM-I + II) than in those with TNM III + IV (p = 0.036). A higher number of tumor-infiltrating lymphocytes (CD4 and CD8) was found in the invasive front of CRC with lymphoid follicles (140.49 ± 12.44 vs 106.13 ± 8.77; p = 0.024 and 215.91 ± 19.17 vs 157.63 ± 11.39; p = 0.015; respectively), Fig. 5A. The same relationship was observed for tumor-associated neutrophils in the tumor center (59.55 ± 5.62 vs 45.49 ± 6.97; p = 0.004), Fig. 5A.

We also analyzed the interrelationships between tumor-infiltrating immune cells (TIICs). Statistically significant correlations were found between the numbers of some TIICs in the tumor center and invasive front. In the tumor center, only CD3-TC was weakly and positively correlated with each cell type assessed, Table 6. In the invasive front, a similar relationship was found for CD4-IF. Other relationships were found for neutrophils. At the tumor center, the number of neutrophils (TAN-TC) was weak and positively correlated only with the number of CD3-TC, while the number of neutrophils in the invasive front (TAN-IF) was weak and positively correlated only with the number of CD4-IF. The number of neutrophils in the invasive front (TAN-IF) was weak and positively correlated (r = 0.36) with the number of neutrophils in the tumor center (TAN-TC). Similarly, a weak and positive correlation at both locations (TC vs IF) was found for CD3 (r = 0.20), CD4 (r = 0.21), and CD8 (r = 0.39).

**Table 6:** Spearman's rank correlation coefficients between selected tumor-infiltrating immune cells.

	CD8 TC	CD8 IF	CD4 TC	CD4 IF	CD3 TC	CD3 IF	TAN TC	TAN IF
CD8 TC	1.000	0.448*	0.167*	0.129	0.207*	0.130	0.158*	0.047
CD8 IF	0.448*	1.000	0.128	0.231*	0.116	0.247*	0.162*	0.035
CD4 TC	0.167*	0.128	1.000	0.394*	0.434*	0.200*	0.053	0.183*
CD4 IF	0.129	0.231*	0.394*	1.000	0.201*	0.298*	0.185*	0.195*
CD3 TC	0.207*	0.116	0.434*	0.201*	1.000	0.517*	0.141*	0.281*
CD3 IF	0.130	0.247*	0.200*	0.298*	0.517*	1.000	0.056	0.124
TAN TC	0.158*	0.162*	0.053	0.185*	0.141*	0.056	1.000	0.442*
TAN IF	0.047	0.035	0.183*	0.195*	0.281*	0.124	0.442*	1.000

Marked correlation coefficients are significant with p < 0.05.

Some differences were found depending on the patients' sex. The number of CD3 lymphocytes was higher in men both in the tumor center and in the invasive front (36.47 ± 4.35 vs. 30.47 ± 4.35; p = 0.00; 149.70 ± 13.77 vs. 107.13 ± 12.00; p = 0.0495, respectively), Fig. 5B.

The presence of lymphoid follicles positively correlated with the density of some tumor-infiltrating immune cells in the tumor center (CD8, TAN) and with the density of some tumor-infiltrating lymphocytes (CD8) in the invasive front, Table 7. Among tumor-infiltrating immune cells, only tumor-associated neutrophils correlated with one of the budding parameters. When analyzing this relationship, it was found that the number of tumor-associated neutrophils in the tumor center (TAN-TC) was higher in the CRC with POR-II + III than in POR-I (58.72 ± 7.33 vs. 46.49 ± 5.26; p = 0.024). It seems that both indicators are independent of each other.

**Table 7:** Spearman's rank correlation coefficients between selected tumor-infiltrating immune cells and tumor budding parameters and lymphoid follicles.

	CD8 TC	CD8 IF	CD4 TC	CD4 IF	CD3 TC	CD3 IF	TAN TC	TAN IF
TBF	− 0.062	− 0.105	0.139	0.034	− 0.009	0.021	− 0.045	0.075
PDC	0.062	− 0.086	0.021	− 0.052	− 0.036	− 0.033	0.122	− 0.135
POR	0.066	− 0.041	0.017	− 0.050	0.009	− 0.036	0.163*	− 0.099
LF	0.192*	0.196*	− 0.035	0.131	0.017	0.096	0.282*	0.139

Marked correlation coefficients are significant with p < 0.05.

Combining the above information, it can be concluded that high tumor-infiltrating immune cells density and high lymphoid follicles density were observed in cases of low-advanced cancer assessed by TNM stage.

## Discussion

CRC is characterized by high intratumor heterogeneity; therefore, many parameters are assessed to determine the advancement of cancer, eligibility of patients for the most effective treatment, and risk stratification. For this purpose, TNM classification is widely used, but it is already known that it is not perfect, as an increase in the TNM stage does not always go hand in hand with a worse prognosis. As a result, qualifying for the best therapy based solely on TNM classification may not be the best choice.

With the widespread use of colonoscopy screening programs, the number of CRC patients diagnosed with low- and intermediate-stage disease is increasing worldwide. The estimation of such patients for optimal treatment is becoming increasingly important; therefore, additional indicators are needed to better assess the prognosis in these patients^9^.

Tumor budding is a histological manifestation of epithelial-mesenchymal transition, initiating infiltration and metastasis in the tumor invasive front. Most of our patients had locally advanced neoplastic disease with a T3 depth of CRC invasion (77.95%) and a moderately G2 differentiated tumor (88.14%). Distant metastases were found in a minority (26.15%) of patients, but regional lymph node involvement was observed in the majority of cases (59.79%). Most of the patients (33.68%) had stage II CRC according to TMN and therefore belonged to a very heterogeneous group in terms of disease progression and outcome, with reported 5-year survival rates ranging from 32 to 66%^14^.

The lowest ratio of isolated malignant cells or clusters in the stroma (TBF-1) was found in the vast majority (75.38%) of patients, while the lowest count of poorly differentiated cancer cell clusters (PDC-1) and the lowest count of poorly differentiated component (POR-1) were found in almost half of the patients at 46.39% and 48.45%, respectively. In other studies, the distribution of budding severity was similar to that in our study^15–17^.

The analysis of tumor budding parameters (TBPs) and classical TNM parameters of CRC severity showed interesting results. All assessed TBPs in our patients were strongly correlated with each other, with the severity of malignancy, the presence and severity of lymph node metastasis, and the presence of distant metastases. We also showed an evident change in the severity of TBPs with CRC progression. Severe TBF was strongly correlated with the high-grade pathological stage of CRC according to the TNM classification, from stage IIA to stage III. Furthermore, TBF progressively increased as the CRC stage increased from stage IIA to stage IIIC. A similar correlation with the increased stage of CRC was visible for PDC and POR.

Other studies have reported similar observations; tumor budding was positively correlated with nodal status, tumor grade, pT stage, perineural invasion, lymphovascular invasion, lung metastases, and the infiltrative tumor margin^18–22^. There are few data in the literature on the relationship between tumor budding and the age or sex of patients and the diameter, or histological type of cancer. We also found no correlation between TBPs and sex or tumor diameter, but interestingly, in our study, PDC was significantly higher in younger patients (p = 0.038), which can contribute to explaining the problem of more frequent occurrences of tumors in this age group. TBF was also higher in mucinous adenocarcinomas than in nonmucinous adenocarcinomas (p = 0.027), which is in line with research demonstrating the association of a mucinous morphology of colorectal cancer with a lower five-year survival^23^.

Unfortunately, there are no data on long-term outcomes in our patients. However, a number of studies have demonstrated a relationship between high tumor budding and poor overall survival, poor disease-free survival, and tumor recurrence^24–26^. Tumor budding in colorectal cancer (CRC) has been extensively studied and previously recognized as an independent risk factor for an unfavorable outcome. Additionally, the use of tumor budding as a parameter in making treatment decisions has already found its place in some practical recommendations for clinicians regarding endoscopic dissection^27^. The results of our research and the studies of other authors clearly confirm that TBPs are important predictors of CRC advancement. Therefore, the classification of CRC based on TBPs seems to be an interesting and practical complement to the classic TNM classification.

We found no correlation between LFs and sex or age of patients, tumor location, or density of TIICs. The overall incidence of lymphoid follicles (LFs) in CRC was previously estimated to be 27.2%, and the incidence of LFs was slightly higher in females (33.6% vs. 24.9%)^28^. In our study, LFs were found in almost half of patients, and their presence in CRC tissue was higher in patients with lower disease advancement according to the TNM classification. This finding is in line with some studies showing a better prognosis in patients with LFs compared with those without LFs^29–31^. In addition, in other studies, LFs were correlated with the densities of intratumoral and peritumoral T cells, and a high LFs density was a marker of better survival^32,33^. Taken together, it appears that lymphoid follicles may reflect host physical defense against CRC and be an indicator of a favorable prognosis.

In our study, a decidedly higher number of tumor-infiltrating immune cells (TIICs) was found in the invasive front than in the tumor center. The composition and density of individual cells at different locations within the tumor varied. The highest density was found for CD8 cells in the invasive front, while the lowest density was found for CD4 cells in the tumor center. Additionally, in another study, the incidence of tumor-infiltrating lymphocytes was higher in the invasive front than in the tumor center, although the incidence of individual lymphocytes was different and CD3 cells were observed relatively most frequently^34^.

We found some differences in the tumor infiltrating-lymphocytes (TILs) density in the primary tumor depending on the stage of cancer and its clinicopathological features. We observed a lower CD8 cell density in the invasive front in CRC patients with distant metastases than in those without metastases. This may be due to a change in the phenotype of the cancer cells over time, before they start to metastasize. Similar observations were made by other authors who showed that high densities of tumor-infiltrating CD8 + cells are associated with improved disease-free and overall survival in CRC, and the high immune cell infiltration by cytotoxic CD8 + T-cells has a favorable prognostic significance^35^. Additionally, it was shown that the presence of high infiltrations of CD3 +, CD8 + lymphocytes in the tumor margins was associated with TNM stages I-II and the absence of lymph node metastases^36^.

In the invasive front, we found a higher density of CD3 and CD4 cells in less advanced cancers, i.e., in T1-T2 CRC and in lower TNM stage (TNM-I vs TNM-II and TNM-I vs TNM-III). The results of another study showed that patients with stage T4 disease had higher expression of CD8 cells in the tumor center than those with other T stages^37^. Other authors, in turn, believe that the immune response in advanced (metastatic) CRC does not appear to influence survival, in contrast to early-stage CRC^38^.

Our findings confirm the observations made thus far by other authors. A recent analysis has suggested that high total TILs counts and CD3 cell density have the strongest association with a survival benefit in CRC patients with regard to longer disease-free survival, cancer-specific survival, and overall survival^39^. According to other authors, TILs in the invasive margin and CD8 cell density in the tumor center may be prognostic factors^34^.

Older people have (as is known) different immunity than younger people. In our patients, the density of lymphocytes in the tumor tissue was different in the elderly group than in the younger group. However, the age-related change did not affect all cells assessed, but only one type and one location within the tumor. In people aged 70 years and above, we found increased expression of CD3 cells in the invasive front. However, it is not known whether the immune function of CD3 cells was altered. Different observations have been made by other authors, who observed a lower expression of both CD3 and CD8 cells in the invasive front in patients aged 75 years or older^37^. This finding requires further research and clarification.

Since it was found that the half-life of human neutrophils reaches more than 5 days, which seems to be sufficient time to allow bioactive molecules released from this type of cell to act in the tumor microenvironment, there has been an increased interest in tumor-associated neutrophils at different intratumoral subsites^40^. One study found that the TAN density in the invasive front showed an inverse relationship with the CRC stage, but no such relationship was found for the TAN density in the tumor center^41^. Another study also showed that high levels of TANs were associated with a low/moderate tumor grade and a nearly threefold increase in overall survival^42^. In one study, the density of TANs was high in low and medium CRC stages (I–III) and dramatically decreased in stage IV disease, suggesting that neutrophil infiltration is a dynamic process that evolves during the course of tumor progression^43^. In our study, we found a similar clinical significance. In the invasive front, we observed a higher density of TANs in lower TNM stage CRC (TNM-I vs TNM-III and TNM-I vs TNM-IV, TNM I + II vs TNM III + IV). Our findings suggest that local immunity may inhibit the progression of CRC, and its assessment may contribute to the goal of optimizing the tumor classification and cancer staging to fit and plan the best therapy.

## Conclusions

Higher tumor budding parameters were observed in high-advanced CRC. The presence of lymphoid follicles and the densities of tumor-infiltrating lymphocytes and tumor-associated neutrophils in the invasive front were lower in more advanced CRCs.

The three histopathology markers assessed within colorectal cancer, such as high tumor budding, scanty lymphocyte infiltration, and the poverty of lymphoid follicles, appear to be reliable indicators of cancer higher staging and progression. These parameters complement each other, and their combined assessment may prove very helpful in predicting prognosis and qualifying patients for the best treatment, but their widespread use requires further research. We propose to take into account histopathology markers in the assessment of colorectal cancer advancement.

## Supplementary Information

Supplementary Information.

## Data Availability

The dataset used during the current study is available as Supplementary Data File.
