# Supplementary material for: Simultaneous analysis of tumor-infiltrating immune cells density, tumor budding status, and presence of lymphoid follicles in CRC tissue
Source: Sci Rep. 2022 Dec 16;12:21732. doi: 10.1038/s41598-022-26225-8 (PMC9758132; doi:10.1038/s41598-022-26225-8)
Supplement: Supplementary file 1 — Supplementary Information. [file 41598_2022_26225_MOESM1_ESM.pdf]

NO	CD8TC	CD8IF	CD4TC	CD4IF	CD3TC	CD3IF	TANTC	TANIF	AGE
**1**	20	100	30	130	30	31	40	35	55
**2**	28	120	29	110	20	60	30	120	61
**3**	27	96	20	98	110	507	77	23	83
**4**	30	130	40	140	30	300	20	40	66
**5**	40	200	11	208	20	270	24	17	67
**6**	312	559	7	359	36	160	224	128	71
**7**	20	210	60	320	38	150	160	120	58
**8**	30	160	40	210	49	210	140	130	72
**9**	20	150	30	180	50	180	90	180	70
**10**	70	1180	38	190	5	323	25	30	50
**11**	61	110	20	170	9	270	29	80	70
**12**	90	120	130	280	10	39	40	90	72
**13**	90	170	160	290	40	180	50	88	71
**14**	90	180	90	190	48	160	80	100	60
**15**	49	170	2	61	38	190	5	8	57
**16**	81	350	60	120	9	242	40	60	73
**17**	93	210	90	110	120	150	90	120	67
**18**	81	340	10	383	1	89	9	66	60
**19**	41	165	2	79	41	256	2	5	59
**20**	609	1005	41	90	182	162	80	9	54
**21**	50	130	30	90	190	201	30	40	73
**22**	65	140	29	98	190	230	38	29	63
**23**	70	210	40	90	60	230	33	46	75
**24**	30	270	30	90	40	323	45	52	51
**25**	90	130	39	53	48	310	46	17	83
**26**	190	120	49	110	18	60	40	22	67
**27**	120	190	39	99	12	89	40	98	84
**28**	133	270	40	190	30	90	32	89	69
**29**	19	80	42	90	26	89	33	99	68
**30**	99	199	45	69	13	90	23	89	79
**31**	81	60	11	22	33	204	4	90	69
**32**	64	180	43	99	23	189	5	90	70
**33**	199	441	56	96	28	160	14	22	81
**34**	40	419	67	110	34	178	24	33	82
**35**	163	754	20	169	28	160	32	12	66
**36**	19	190	47	89	34	280	19	78	67
**37**	59	104	18	72	8	201	68	7	72
**38**	19	99	70	190	17	189	18	89	71
**39**	47	242	34	78	22	16	64	27	79
**40**	29	89	12	120	33	100	98	110	75
**41**	22	88	28	39	48	130	88	160	73
**42**	888	190	29	230	55	180	66	180	76
**43**	15	127	3	3	13	517	108	161	75
**44**	22	190	43	89	3	86	126	23	69
**45**	21	98	32	89	11	89	37	120	27
**46**	10	190	20	87	33	98	17	89	71
**47**	19	90	31	89	25	99	19	160	68
**49**	111	580	41	36	44	89	133	20	78
**50**	20	384	44	89	99	20	170	290	71
**52**	2	64	4	5	3	18	14	17	80
**54**	18	623	5	132	22	244	13	23	50
**55**	23	105	13	207	20	148	47	33	76

**56**	174	219	0	247	56	189	157	13	78
**57**	91	120	0	159	13	58	85	1	70
**58**	72	489	26	669	11	1125	43	19	71
**59**	89	150	34	187	33	180	160	230	59
**60**	88	160	33	178	66	185	36	320	75
**61**	102	180	19	89	33	170	43	0	72
**63**	54	190	60	140	38	150	22	67	63
**64**	16	89	55	87	32	79	15	7	54
**65**	19	34	1	2	2	9	37	5	51
**66**	13	92	0	101	0	66	84	33	61
**67**	79	165	0	0	0	15	28	15	47
**68**	15	360	33	90	54	120	54	170	79
**69**	5	77	0	109	0	113	47	19	57
**70**	88	290	49	99	35	120	46	89	55
**71**	99	185	28	90	180	320	29	22	75
**72**	25	188	18	2	38	89	23	68	72
**73**	78	99	26	59	2	2	29	0	65
**74**	90	290	50	321	1	3	14	3	47
**75**	54	49	81	99	37	138	16	37	89
**76**	9	90	0	11	4	54	16	5	68
**77**	13	133	12	9	12	9	18	5	77
**78**	34	133	0	34	89	190	25	58	68
**79**	4	236	99	175	80	150	4	15	83
**80**	36	97	0	0	1	16	7	8	75
**81**	3	158	190	140	37	180	30	23	81
**82**	6	112	60	99	78	190	14	29	67
**83**	58	275	1	37	29	519	141	341	70
**84**	55	202	2	54	60	160	2	11	73
**85**	4	37	0	0	0	18	14	12	77
**86**	29	88	77	149	137	219	19	88	75
**87**	26	99	39	147	44	167	39	140	72
**88**	18	25	67	160	9	14	18	12	48
**89**	31	374	20	190	29	230	298	1031	55
**90**	65	178	20	10	0	0	38	14	60
**91**	191	1	1	0	0	1	137	45	71
**92**	67	347	2	60	6	701	22	60	70
**93**	32	68	11	78	29	160	50	82	61
**94**	17	65	0	0	1	2	3	2	50
**95**	27	284	0	501	0	41	19	5	72
**96**	65	190	34	211	0	16	18	4	51
**97**	45	219	67	230	0	6	178	4	65
**98**	19	134	67	160	320	18	89	150	43
**99**	69	147	17	0	1	25	17	35	75
**100**	58	90	22	159	2	37	23	10	65
**101**	280	405	45	6	52	71	108	136	69
**102**	32	185	0	13	0	238	22	4	77
**103**	9	61	0	6	90	130	3	16	64
**104**	43	170	150	270	19	89	6	76	51
**105**	35	254	4	69	0	4	6	1	67
**106**	39	313	55	99	9	140	27	4	52
**107**	26	260	0	2	0	11	123	26	67
**108**	6	158	0	0	0	269	35	17	48
**109**	0	104	0	65	2	53	11	0	88

**110**	0	11	34	98	0	2	2	1	67
**111**	127	76	0	47	10	56	35	248	66
**112**	41	300	2	170	29	446	41	7	77
**113**	18	24	0	67	2	8	8	7	81
**114**	17	28	18	67	88	36	67	52	74
**115**	30	383	31	73	10	69	9	219	69
**116**	35	325	90	180	6	20	44	205	76
**117**	28	160	0	61	1	38	49	93	70
**118**	18	296	0	1	6	20	37	105	70
**119**	63	405	0	241	9	286	61	87	79
**120**	12	11	0	48	0	2	49	241	62
**121**	15	162	55	190	74	255	9	4	74
**122**	30	115	31	243	60	140	118	104	75
**123**	33	106	33	89	25	87	4	0	64
**124**	11	138	19	514	6	331	29	45	75
**125**	16	178	19	486	35	67	21	68	47
**126**	122	469	0	0	13	124	144	46	64
**127**	15	390	0	2	0	0	77	89	58
**128**	2	64	29	89	6	197	8	10	70
**129**	5	14	0	1	1	2	16	27	53
**130**	18	89	15	160	20	190	80	190	55
**131**	27	76	8	398	88	219	86	673	43
**132**	27	169	0	237	30	357	29	22	39
**133**	90	190	76	169	30	89	34	23	32
**134**	80	189	20	230	45	310	23	178	70
**135**	64	191	0	0	99	180	3	13	70
**136**	87	69	88	90	180	29	93	70	70
**137**	209	83	7	34	15	33	9	81	71
**138**	101	247	1	26	3	4	635	9	72
**139**	20	69	22	89	33	140	88	98	74
**140**	1	49	1	36	0	52	14	67	76
**141**	33	78	22	99	11	88	43	98	76
**142**	28	99	17	96	12	89	33	89	77
**143**	22	90	16	120	32	78	22	99	78
**144**	16	68	17	88	0	12	45	64	82
**145**	19	134	55	99	12	78	56	134	85
**146**	19	88	67	98	43	79	73	140	57
**147**	78	386	13	226	11	43	127	539	58
**148**	55	190	33	154	27	32	48	123	62
**149**	87	178	43	167	34	78	39	143	67
**150**	11	79	18	58	1	31	7	15	74
**152**	89	189	16	56	23	167	18	29	35
**153**	77	159	23	79	12	59	89	99	54
**154**	19	88	90	20	180	340	78	89	61
**155**	3	193	4	213	0	2	84	13	64
**156**	6	33	2	9	5	1	16	14	66
**157**	75	228	5	187	43	140	12	10	50
**158**	94	290	7	49	76	98	18	60	52
**159**	79	319	65	100	19	99	31	4	58
**160**	44	312	24	78	27	87	65	125	61
**161**	27	89	19	120	16	78	55	170	67
**162**	52	96	15	85	89	140	21	29	71
**163**	0	407	0	133	0	366	26	18	72

**164**	155	230	19	189	0	0	39	176	84
**165**	18	135	28	340	20	180	32	167	68
**166**	7	37	34	99	67	160	170	350	68
**167**	126	268	24	579	130	361	174	22	72
**168**	23	189	55	167	30	342	4	1	71
**169**	20	111	34	88	12	88	11	9	74
**170**	62	62	7	169	22	67	83	9	74
**172**	47	80	90	65	32	140	15	9	77
**173**	104	71	45	99	25	190	9	1	83
**174**	32	188	13	88	67	19	15	10	79
**175**	44	99	34	120	45	130	18	89	83
**176**	53	430	90	170	90	160	43	3	63
**177**	86	161	0	93	6	115	44	9	41
**178**	78	90	30	120	88	140	49	89	52
**179**	9	78	45	140	28	123	78	167	52
**180**	44	82	9	5	1	48	31	4	53
**181**	36	124	9	95	6	56	46	1	65
**182**	32	145	26	160	56	67	123	167	61
**183**	89	176	17	52	23	17	2	26	71
**184**	55	145	32	164	33	22	15	160	71
**185**	5	90	4	265	1	50	97	63	66
**186**	89	167	22	230	21	178	23	187	71
**187**	32	45	22	145	34	156	156	230	67
**188**	104	259	1	6	1	2	43	19	72
**189**	22	122	3	97	0	35	138	376	72
**190**	34	67	33	130	34	59	22	140	81
**191**	28	89	11	130	44	78	22	89	77
**192**	294	651	32	134	33	89	29	29	77
**193**	56	230	45	134	23	79	45	120	76
**194**	33	134	23	37	45	89	34	145	76
**195**	56	123	45	148	44	56	26	45	80
**196**	49	300	4	47	45	123	87	403	77
**197**	134	229	0	3	5	29	34	167	58
**198**	35	138	0	193	1	23	11	25	71
**199**	18	147	2	69	8	53	200	22	75
**200**	6	37	2	6	0	33	23	10	69
**201**	2	12	2	150	0	2	23	15	69

SEX	LOCAL	G	T	N	M	TNM	TBF	PDC	POR
M	1	2	1	0	0	I	1	1	1
M	1	2	3	2a	0	III	1	3	3
M	2	2	1	0	0	I	1	2	3
M	2	2	1	0	0	I	1	1	1
K	1	2	3	1	0	III	1	3	2
K	3	2	3	0	0	II	1	1	1
M	1	2	2	0	1	IV	2	2	3
M	1	2	2	0	0	I	1	1	1
K	1	2	2	0	0	I	1	1	1
K	3	2	3	0	0	II	1	1	1
M	2	2	3	0	0	II	1	3	3
K	4	3	3	0	1	IV	2	3	2
M	3	2	2	0	1	IV	3	2	2
M	1	2	3	0	0	II	1	3	3
K	3	2	3	1	1	IV	3	1	1
K	1	2	2	0	0	I	1	1	1
K	3	3	3	0	0	II	3	3	3
K	1	2	3	0	0	II	1	1	1
M	2	2	2	0	1	IV	2	2	2
M	3	2	4	1	0	III	1	2	2
M	3	3	3	0	1	IV	1	2	2
M	2	2	2	0	0	I	1	1	1
M	1	2	2	0	0	I	1	3	3
M	4	2	2	0	0	I	1	1	1
M	3	2	2	0	1	IV	2	2	2
M	3	2	3	0	0	II	2	2	2
M	3	3	3	0	1	IV	3	2	2
K	2	3	3	0	1	IV	3	3	3
M	3	2	2	0	0	I	1	3	3
K	3	3	3	0	0	II	3	2	3
M	3	2	3	0	1	IV	1	1	1
M	1	3	3	0	1	IV	2	2	2
M	1	3	3	0	1	IV	1	2	2
M	1	2	3	2a	0	III	1	1	1
M	2	2	3	0	0	II	2	2	2
M	1	2	2	0	0	I	1	1	1
K	3	2	3	1	0	III	1	3	2
K	1	2	3	0	0	II	1	2	1
M	1	2	3	0	0	II	1	1	1
M	3	2	2	0	0	I	1	1	1
M	1	2	2	0	0	I	1	2	1
K	3	2	2	0	0	I	1	1	2
M	3	2	3	0	0	II	1	1	1
M	3	3	3	0	1	IV	2	3	3
M	1	3	3	0	1	IV	3	2	2
M	3	3	3	0	0	II	1	1	1
K	1	2	2	0	0	I	1	1	1
M	2	2	3	0	0	II	1	1	1
M	2	3	2	1	0	III	1	2	2
K	2	2	4	1	1	IV	2	3	3
M	1	2	4	0	0	III	1	1	1
K	1	2	3	1	0	III	1	1	1

M	2	2	3	0	0	III	1	2	2
M	2	2	3	1	0	III	1	2	1
M	1	2	2	0	0	I	1	1	1
K	3	2	3	0	1	IV	2	2	1
K	3	2	2	0	0	I	1	1	1
K	1	2	2	2a	0	III	1	3	3
K	1	2	4	1	1	IV	2	1	1
M	1	2	3	0	1	IV	2	2	2
K	2	2	3	2a	1	IV	2	2	2
K	1	2	3	0	1	IV	2	2	2
K	3	3	3	2a	1	IV	2	2	2
M	3	3	3	0	1	IV	2	2	2
M	2	2	3	1	1	IV	2	2	2
M	3	2	3	2b	1	IV	2	2	2
K	4	2	3	0	1	IV	1	3	3
M	4	2	3	1	1	IV	2	2	2
K	2	2	2	0	0	I	1	1	1
M	1	2	2	0	0	I	1	1	1
M	3	2	3	0	0	II	1	1	1
K	2	2	3	2a	0	III	1	2	2
M	1	2	3	1	1	IV	1	2	2
M	3	2	3	1	0	III	1	1	1
K	2	2	3	1	0	III	1	1	1
M	1	2	3	1	0	III	1	1	1
K	3	2	3	1	0	III	1	1	1
M	1	2	3	0	0	II	1	1	1
M	1	2	2	0	0	I	1	1	1
M	1	2	3	0	0	II	1	2	2
M	1	2	3	0	0	II	1	2	1
K	1	2	3	0	0	II	1	1	1
K	2	2	3	0	0	II	1	1	1
K	2	2	3	2b	0	III	1	1	1
M	3	2	3	0	0	II	1	1	1
K	1	2	3	0	0	II	1	2	2
M	3	2	3	0	1	IV	1	1	1
K	4	2	3	1	0	III	1	2	2
K	2	2	3	0	0	II	1	1	2
K	2	2	3	1	0	III	1	3	3
K	2	2	3	1	0	III	1	1	1
M	1	2	3	1	0	III	1	3	3
K	1	2	3	0	0	II	1	2	2
M	1	2	3	0	0	II	1	1	1
M	1	2	3	1	0	III	1	2	1
K	2	2	3	2b	0	III	1	3	2
M	3	2	3	2b	0	III	1	3	3
M	2	2	3	1	0	III	1	1	1
K	2	2	3	1	1	IV	2	1	1
M	2	2	3	2a	0	III	1	2	2
K	2	2	3	2a	0	III	1	1	1
M	2	2	3	0	0	II	1	1	1
K	1	2	3	0	0	II	1	1	1
M	3	2	3	0	0	II	1	2	1
K	3	2	3	1	0	III	1	1	1

M	1	2	3	0	1	IV	2	1	1
K	1	2	3	0	0	II	1	1	1
M	1	2	3	0	0	II	1	1	1
M	1	2	3	0	0	II	1	1	1
K	1	2	3	1	0	III	1	3	3
M	2	2	3	1	0	III	1	1	1
K	1	2	3	0	0	II	1	1	1
K	3	2	3	0	0	II	1	2	3
K	4	2	3	1	0	III	1	1	1
M	4	2	3	0	0	II	1	1	1
K	1	2	3	0	1	IV	2	3	3
M	2	2	3	2a	1	IV	3	2	2
M	4	2	3	1	0	III	1	2	2
M	1	2	3	2b	0	III	1	3	3
K	2	2	3	0	0	II	1	1	1
K	2	2	3	0	0	II	1	1	1
K	3	2	3	0	0	II	1	2	2
K	1	2	3	1	0	III	1	1	1
M	1	2	3	0	0	II	1	1	1
M	1	2	4	1	0	III	1	1	1
M	1	3	3	2	1	IV	3	2	2
M	1	2	2	0	0	I	1	3	3
M	3	3	3	0	0	II	1	2	2
K	1	2	2	0	0	I	1	1	1
M	1	2	3	1	1	IV	3	1	1
K	2	2	3	0	0	II	1	2	1
K	1	2	4	2b	0	III	1	3	3
K	1	2	2	0	0	I	1	1	1
M	4	2	3	0	0	II	1	3	3
M	3	3	4	2b	1	IV	3	1	1
K	1	2	3	2a	1	IV	2	3	3
M	4	2	3	1	0	III	1	1	1
M	4	3	3	2a	1	IV	2	2	2
K	2	2	3	1	1	IV	2	2	2
K	2	2	3	0	1	IV	2	1	1
M	2	2	3	0	0	II	1	2	2
K	1	2	3	0	0	II	1	2	2
M	1	2	3	0	0	II	1	1	1
K	4	3	3	1	0	III	1	2	2
K	2	2	3	0	0	II	1	1	1
M	3	3	3	0	0	II	1	2	2
K	1	2	3	1	0	III	1	1	1
K	2	1	1	0	0	I	1	1	1
M	1	2	3	2b	1	IV	2	2	2
K	2	2	3	1	0	III	1	2	2
M	3	2	3	2b	0	III	1	2	2
M	1	2	3	2a	1	IV	1	1	1
K	4	2	4	0	0	II	1	1	1
K	1	2	3	0	1	IV	2	3	2
M	2	2	1	0	0	I	1	2	2
M	1	2	3	1	0	III	1	1	1
K	1	2	3	0	0	II	1	3	3
M	1	2	3	1	1	IV	2	3	3

M	3	2	3	1	1	IV	2	2	2
M	1	3	3	1	0	III	2	2	2
K	2	2	3	1	0	III	1	2	1
M	4	2	3	1	1	IV	1	3	3
M	1	2	3	0	0	II	1	3	3
K	3	2	3	0	0	II	1	1	1
M	2	2	3	1	0	III	1	2	2
M	2	2	3	0	1	IV	2	3	3
M	1	2	2	0	0	I	1	2	2
M	1	2	3	0	0	II	1	1	2
M	3	2	3	1	1	IV	2	1	1
M	3	2	3	0	0	II	1	2	2
M	2	3	3	0	0	II	1	3	3
M	2	2	3	1	0	III	1	2	2
K	2	2	3	2b	1	IV	2	1	1
M	2	2	3	0	0	II	1	2	2
M	2	2	3	0	0	II	1	1	2
K	1	2	3	1	0	III	1	1	1
M	1	2	3	0	0	II	1	2	1
M	1	2	3	1	1	IV	2	1	1
M	1	2	4	2b	0	III	1	3	3
K	1	2	4	2b	0	III	1	1	1
M	1	2	3	1	0	III	1	2	2
K	3	2	4	0	0	II	1	2	2
M	1	2	3	0	0	II	1	3	3
K	1	2	3	0	0	II	1	1	1
M	3	2	3	1	0	III	1	1	1
M	3	2	3	0	0	II	1	1	1
M	2	2	3	1	0	III	1	1	1
M	1	2	3	1	0	III	1	2	2
K	2	2	3	1	1	IV	2	1	1
K	4	2	3	2a	0	III	1	1	3
M	3	2	3	0	0	II	1	2	2
M	1	2	3	0	0	II	1	1	1
M	1	2	3	0	0	II	1	2	2
M	1	2	3	0	0	II	1	1	2
K	2	2	3	1	1	IV	2	3	2
